# Gut bacterial microbiota in patients with myasthenia gravis: results from the MYBIOM study

**DOI:** 10.1177/17562864211035657

**Published:** 2021-08-11

**Authors:** Andreas Totzeck, Elakiya Ramakrishnan, Melina Schlag, Benjamin Stolte, Kathrin Kizina, Saskia Bolz, Andreas Thimm, Mark Stettner, Julian R. Marchesi, Jan Buer, Christoph Kleinschnitz, Hedda Luise Verhasselt, Tim Hagenacker

**Affiliations:** Department of Neurology and Center for Translational Neuro- and Behavioral Sciences (C-TNBS), University Hospital Essen, Hufelandstr 55, Essen, 45147, Germany; Department of Neurology and Center for Translational Neuro- and Behavioral Sciences (C-TNBS), University Hospital Essen, University of Duisburg-Essen, Essen, Germany; Department of Neurology and Center for Translational Neuro- and Behavioral Sciences (C-TNBS), University Hospital Essen, University of Duisburg-Essen, Essen, Germany; Department of Neurology and Center for Translational Neuro- and Behavioral Sciences (C-TNBS), University Hospital Essen, University of Duisburg-Essen, Essen, Germany; Department of Neurology and Center for Translational Neuro- and Behavioral Sciences (C-TNBS), University Hospital Essen, University of Duisburg-Essen, Essen, Germany; Department of Neurology and Center for Translational Neuro- and Behavioral Sciences (C-TNBS), University Hospital Essen, University of Duisburg-Essen, Essen, Germany; Department of Neurology and Center for Translational Neuro- and Behavioral Sciences (C-TNBS), University Hospital Essen, University of Duisburg-Essen, Essen, Germany; Department of Neurology and Center for Translational Neuro- and Behavioral Sciences (C-TNBS), University Hospital Essen, University of Duisburg-Essen, Essen, Germany; Department of Metabolism, Digestion and Reproduction, Imperial College London, London, UK; Institute of Medical Microbiology, University Hospital Essen, University of Duisburg-Essen, Essen, Germany; Department of Neurology and Center for Translational Neuro- and Behavioral Sciences (C-TNBS), University Hospital Essen, University of Duisburg-Essen, Essen, Germany; Institute of Medical Microbiology, University Hospital Essen, University of Duisburg-Essen, Essen, Germany; Department of Neurology and Center for Translational Neuro- and Behavioral Sciences (C-TNBS), University Hospital Essen, University of Duisburg-Essen, Essen, Germany

**Keywords:** CIDP, MG, Deltaproteobacteria, *Faecalibacterium*

## Abstract

**Background:**

Myasthenia gravis (MG) is an autoimmune neuromuscular disease, with gut microbiota considered to be a pathogenetic factor. Previous pilot studies have found differences in the gut microbiota of patients with MG and healthy individuals. To determine whether gut microbiota has a pathogenetic role in MG, we compared the gut microbiota of patients with MG with that of patients with non-inflammatory and inflammatory neurological disorders of the peripheral nervous system (primary endpoint) and healthy volunteers (secondary endpoint).

**Methods:**

Faecal samples were collected from patients with MG (*n* = 41), non-inflammatory neurological disorder (NIND, *n* = 18), chronic inflammatory demyelinating polyradiculoneuropathy (CIDP, *n* = 6) and healthy volunteers (*n* = 12). DNA was isolated from these samples, and the variable regions of the *16S rRNA* gene were sequenced and statistically analysed.

**Results:**

No differences were found in alpha- and beta-diversity indices computed between the MG, NIND and CIDP groups, indicating an unaltered bacterial diversity and structure of the microbial community. However, the alpha-diversity indices, namely Shannon, Chao 1 and abundance-based coverage estimators, were significantly reduced between the MG group and healthy volunteers. Deltaproteobacteria and *Faecalibacterium* were abundant within the faecal microbiota of patients with MG compared with controls with non-inflammatory diseases.

**Conclusion:**

Although the overall diversity and structure of the gut microbiota did not differ between the MG, NIND and CIDP groups, the significant difference in the abundance of Deltaproteobacteria and *Faecalibacterium* supports the possible role of gut microbiota as a contributor to pathogenesis of MG. Further studies are needed to confirm these findings and to develop possible treatment strategies.

## Introduction

Myasthenia gravis (MG) is an autoimmune-mediated disorder in which autoantibodies target different proteins of the neuromuscular junction, particularly the acetylcholine receptor (AChR). The pathogenetic pathway of MG is T cell and B cell dependent. Regulatory T cells (Treg) and CD4^+^ T cells recognise AChR epitopes and trigger B cells to produce antibodies.^1–4^ Foxp3^+^ CD4^+^ Treg cells influence the amount of autoreactive T cells, regulate the activity of autoreactive B cells, and therefore regulate the production of AChR antibodies.^5^ In patients with MG, the frequency of Foxp3^+^ CD4^+^ Treg cells is significantly lowered, contributing to the pathogenesis of MG.^6–8^ However, the predisposing factors for MG are still unknown. Foxp3^+^ CD4^+^ Treg cells occur more frequently in the colonic lamina propria compared with other organs and are considered to maintain homeostasis of the gut microbiota.^9–11^ Perturbations in the composition of gut microbiota can lead to Foxp3^+^ CD4^+^ Treg cell deficiency. Gut microbiota profiles of patients with MG seem to differ from those of healthy volunteers.^12,13^ However, data on gut microbiota profiles of patients with MG compared with those of patients with other inflammatory or non-inflammatory neurological diseases are still lacking. Therefore, we set up an observational study with the primary endpoint of determining whether the gut microbiota is altered in patients with MG compared with patients with non-inflammatory neurological disorders (NINDs) and patients with chronic inflammatory demyelinating polyradiculoneuropathy (CIDP), another autoimmune neurological disease. As a secondary endpoint, we compared gut microbiota profiles of patients with MG with those of healthy volunteers without neurological disorders.

## Methods

### Patient recruitment and study population

In a single-centre observational study design, 77 participants were recruited *via* the Department of Neurology at the University Hospital of Essen, Germany, between July 2017 and August 2018. This study, named MYBIOM study (microbiota in MG patients), was approved by the Ethics Committee of the Medical Faculty at the University Duisburg-Essen, Germany (approval number: 17-7439-BO). Written informed consent was obtained from each patient prior to inclusion in the study. MG diagnosis (*n* = 41) was based on the patient’s medical history, clinical characteristics such as fluctuating fatigability and muscle weakness, a positive response to cholinesterase inhibitors and, optionally, recorded decrement in repetitive motor nerve stimulation.^14^ Quantitative myasthenia gravis (QMG) score, the clinical subtype of MG, and the Myasthenia Gravis Foundation of America (MGFA) classification were determined at the time of study inclusion. In the first comparison group, patients with other NINDs, for example stroke, headache or Parkinson’s disease, were recruited (*n* = 18). In the second comparison group, CIDP diagnosis (*n* = 6) was based on the European Federation of Neurological Societies/Peripheral Nerve Society diagnostic criteria.^15^ All patients with CIDP had typical CIDP and no variant.^16^ Furthermore, 12 neurologically healthy volunteers without any underlying neurological or systemic inflammatory disease were recruited as controls. Participants were selected for inclusion in the study if they met the following criteria: ⩾18 years of age, diagnosed MG or any disorder specific for the comparison groups, no history of treatment with antibiotics within the previous 4 weeks, and no intentional consumption of probiotics or anti-obesity agents within 3 months prior to study participation. Further exclusion criteria were: chronic inflammatory bowel disease, short bowel syndrome, irritable bowel syndrome, pregnancy and recent treatment with chemotherapeutics or monoclonal antibodies. Exclusion criteria were designed to reduce confounding effects.

### Sample collection and preparation

Fresh faecal samples were collected from participants in the morning before a visit to the clinics, in the ward (inpatients) or on site, where possible. No drug-induced diarrhoea was present. Faeces were transferred into provided collection tubes using an enclosed spoon and returned to study staff who transported them to the Institute of Medical Microbiology at 4°C within 12 h of specimen collection. Faecal samples were stored at −80°C until DNA isolation.

### DNA extraction and *16S rRNA* gene sequencing

A total of 73 faecal samples, including 6 negative controls (RNA-free water) and 2 positive controls (*Escherichia coli* and a mix of *E. coli*, *Staphylococcus aureus* and *Corynebacterium striatum*) were individually placed in 2 ml tubes prefilled with 1.4 mm ceramic spheres, 0.1 mm silica spheres and 1 4 mm glass bead (Lysing Matrix E, MP Biomedicals Germany, Eschwege, Germany), dissolved in 1 ml InhibitEX buffer (Qiagen, Hilden, Germany) and vortexed until homogenised. A bead-beating step in a MagNA Lyser (Roche Diagnostics, Basel, Switzerland) was applied for 3 × 60 s at 5 m/s with 5 min rest in between. DNA was extracted using QIAmp Fast DNA Stool Mini kit (Qiagen), following the manufacturer’s instructions. Total genomic DNA was eluted in sterile microcentrifuge tubes and quantified using a NanoDrop (Thermo Fisher Scientific, Waltham, MA, USA), following the manufacturer’s instructions. DNA aliquots were stored at −80°C until use. Variable regions of the *16S rRNA* gene were sequenced at BaseClear B.V. (Leiden, The Netherlands). For metataxonomic sequencing, primers F (5′-CCTACGGGNGGCWGCAG-3′) and R (5′-GACTACHVGGGTATCTAATCC-3′)^17^ were used to amplify the V3–V4 regions of the *16S rRNA* gene to generate 10–50 k paired-end reads on an Illumina MiSeq (Illumina, San Diego, CA, USA).

### Illumina demultiplexing

FASTQ sequence read files were generated using bcl2fastq2 v2.18.^18^ Initial quality assessment was based on data passing the Illumina Chastity filtering. Subsequently, reads containing the PhiX control signal were removed using an in-house filtering protocol. In addition, reads containing (partial) adapters were clipped (up to a minimum read length of 50 bp). The second quality assessment was carried out on the remaining reads using the FASTQC quality control tool v0.11.5.^19^ The average number of reads per sample after demultiplexing and filtering was 20,453, and the average yield per sample was 12 Mb.

### Statistical analysis of demographics

Mean, standard error of the mean, median and range were calculated for age, height, weight, body mass index (BMI) and QMG. Data from the MG group were compared with the NIND, CIDP and healthy volunteer groups using the nonparametric Mann–Whitney test.

### Processing and statistical analysis of metataxonomic data

Sequence processing was performed using Mothur v1.43^20^ to reduce possible polymerase chain reaction effects, cluster sequences into operational taxonomic units (OTUs) at the 97% identity cut-off and to provide taxonomic annotations. The Mothur function ‘make.contigs’ was used to join paired-end reads (R1 and R2) for each sample and to trim sequences at the 2.5t–97.5 percentiles on the distribution lengths of the amplicons. Sequences with ambiguities were removed by setting the parameter *N* = 0. Filtered sequences were aligned against the *16S rRNA* reference gene in the SILVA rRNA database (http://www.arb-silva.de). Singleton, nonbacterial and chimera sequences were removed, the latter with the UCHIME tool.^21^ Taxonomic assignment of the processed sequences, from phylum to genus level, was carried out using the Ribosomal Database Project Naïve Bayesian Classifier, using trainset v16 with a cut-off of 80%.^22^ FastTree v2.1.9 has been used to infer approximately maximum-likelihood phylogenetic trees.^23^ Each sample library was subsampled based on the smallest library size to reduce the effect of different sampling methods and to obtain comparable sequencing libraries. OTUs with less than 10 counts were consolidated into a Rare_OTU group to maintain the same number of reads in each sample. Statistical analyses were performed in R v3.2.2 while metataxonomic data analysis was performed using the STAMP tool.^24^

Within-sample alpha-diversity indices [observed OTUs, Chao1, abundance-based coverage estimators (ACEs), Simpson, and Shannon] were calculated in the Mothur software. For comparison between groups, the nonparametric Kruskal–Wallis and Mann–Whitney tests were calculated in GraphPad Prism v7.05 (GraphPad Software, San Diego, CA, USA, www.graphpad.com).

Beta-diversity analysis was performed using log+1 transformed OTU data and principal component analysis (PCA) comparisons of the samples. The permutational analysis of variance approach (PERMANOVA) was undertaken in R using the R Vegan package.

The Spearman distance and Ward’s agglomeration methods were used for hierarchical clustering of faecal samples. Statistical tests with *p* ⩽ 0.05 were considered significant.

### Sequencing metrics

A total of 1,738,487 reads were obtained from sequencing the V3– V4 regions of the *16S rRNA* gene. These were reduced to 1,601,016 reads after quality filtering and 1,084,593 unique sequences were used for the alignment. An average of 9803 reads (5127–19,363) were obtained, per sample, following alignment. Subsampling per library size resulted in >99% average coverage per sample, at 5127 reads per sample. A total of 2152 OTUs were identified, and 367 OTUs with more than 10 counts across samples were retained.

## Results

### Characteristics of the study population

The four groups had similar BMIs. Participants in the NIND and healthy volunteer groups were younger compared with those in the MG group. MG classification at the time of recruitment was as follows: 18 MGFA I, 11 MGFA IIA, 8 MGFA IIB, 3 MGFA IIIA and 1 MGFA IIIB. A total of 36 patients with MG were AChR-antibody positive, and 8 patients had a history of thymectomy (no thymoma). No patients with MG with a history of myasthenic crisis were included in the study. At the time of recruitment, five patients with MG were seronegative but showed abnormal decrement in repetitive motor nerve stimulation and positive reaction to treatment with pyridostigmine according to MG diagnosis.^25^ There was a higher intake of corticosteroids in the MG (prednisone 2.5–70 mg/day) and NIND groups than in the CIDP and healthy volunteer groups. Six patients in the NIND group were taking corticosteroids for asthma, headache or chronic pain. In the MG group, 11 patients were treated with azathioprine (100–400 mg/day), 8 were treated with mycophenolate mofetil (1000–1500 mg/day), and 2 patients were treated with rituximab. In all groups, hypertension and diabetes were among the most frequent comorbidities. All participants were not on a special diet at time of sample collection. Demographic information for study participants is summarised in Table 1. Time of initial diagnosis in patients with MG, NIND and CIDP is illustrated in the supplemental material (Supplemental Table 1).

**Table 1.** Demographics and comorbidities of the MG, NIND, CIDP and healthy volunteer groups.

Characteristic	Group			
	MG	NIND	CIDP	Healthy
Age (years)	64.6 ± 16.2 (68.0; 19–91)	52.0 ± 17.0 (54.0; 25–78)*	57.2 ± 14.6 (62.5; 28–66)	51.4 ± 15.5 (55.5; 22–75)*
Sex (male/female)	24/17	10/8	4/2	4/8
Height (cm)	171.6 ± 9.7 (170.0; 157–196)	172.6 ± 12.2 (172.5; 140–192)	179.7 ± 7.1 (180.0; 169–188)	164.0 ± 5.3 (164.0; 156–175)*
Weight (kg)	81.8 ± 19.0 (85.0; 47–122)	79.0 ± 19.2 (82.0; 36–117)	93.2 ± 20.0 (90.0; 70–126)	71.8 ± 10.2 (70.0; 57–92)
BMI (kg/m²)	27.7 ± 5.7 (27.4; 16.3–41.6)	26.2 ± 4.4 (25.9; 16.0–33.1)	28.8 ± 5.8 (26.4; 24.5–39.8)	26.7 ± 3.4 (27.4; 21.2–32.8)
Clinical subtype				
Ocular	18 (44.0%)	–	–	–
Generalised	23 (56.0%)	–	–	–
MGFA score	I–IIIB			
QMG score	3.9 ± 3.5 (3.5; 0–14)	–	–	–
Corticosteroids intake	24 (58.5%)	6 (33.3%)	1 (16.7%)	0
Other neurological disease				
Chronic back pain/headache (%)	–	8 (44.4)	–	–
Stroke (%)	–	2 (11.1)	–	–
Parkinson’s disease (%)	–	2 (11.1)	–	–
Epilepsy (%)	–	2 (11.1)	–	–
Spinal muscular atrophy (%)	–	2 (11.1)	–	–
Dementia (%)	–	1 (5.6)	–	–
Normal pressure hydrocephalus (%)	–	1 (5.6)	–	–
Comorbidities				
Hypertension (%)	26 (63.4)	6 (33.3)	4 (66.7)	5 (41.7)
Diabetes mellitus (%)	7 (17.1)	2 (11.1)	2 (33.3)	5 (41.7)
Chronic kidney disease (%)	2 (4.9)	1 (5.6)	0	0
Thyroid disease (%)	11 (26.8)	6 (33.3)	1 (16.7)	6 (50.0)
Hyperlipidaemia (%)	7 (17.1)	4 (22.2)	0	3 (25.0)
Coronary heart disease (%)	7 (17.1)	1 (5.6)	0	2 (16.7)
Asthma (%)	3 (7.3)	4 (22.2)	0	1 (8.3)

* Age of NIND *versus* MG: *p* = 0.0089. Age of healthy volunteers *versus* MG: *p* = 0.0120. Height of healthy volunteers *versus* MG: *p* = 0.0136, and CIDP *versus* MG: *p* = 0.0488.

Age, height, weight, BMI and QMG are presented as mean ± standard error of the mean (median; range).

BMI, body mass index; CIDP, chronic inflammatory demyelinating polyradiculoneuropathy; MG, myasthenia gravis; MGFA, Myasthenia Gravis Foundation of America; NIND, non-inflammatory neurological disorder; QMG, quantitative myasthenia gravis.

### Diversity of faecal microbiota

**Figure 1.**
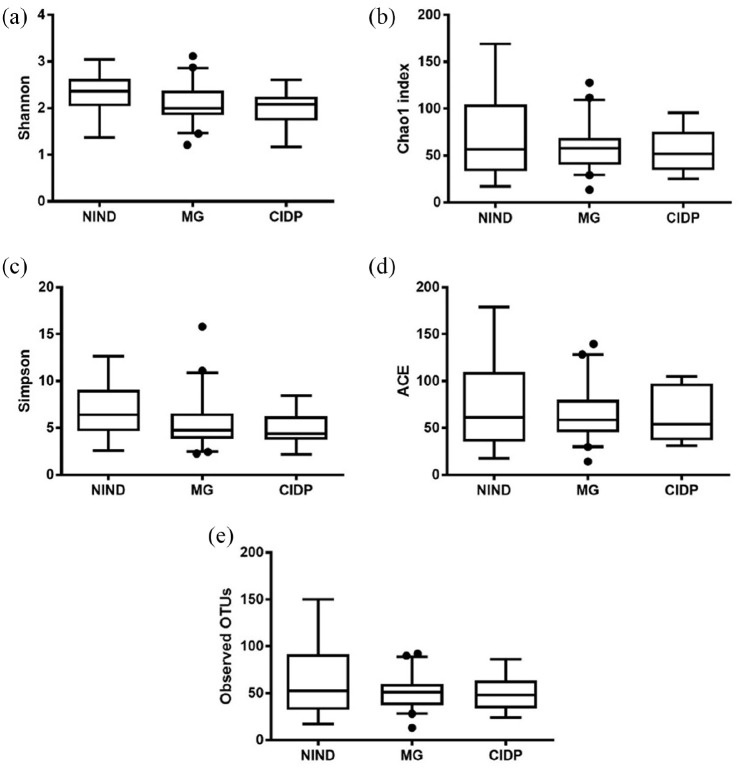
Comparison of alpha-diversity indices of the different study groups MG, NIND and CIDP using the Kruskal–Wallis test. Data are presented as mean ± standard error of the mean. ACE, abundance-based coverage estimator; CIDP, chronic inflammatory demyelinating polyradiculoneuropathy; MG, myasthenia gravis; NIND, non-inflammatory neurological disorder; OTU, operational taxonomic unit.

**Figure 2.**
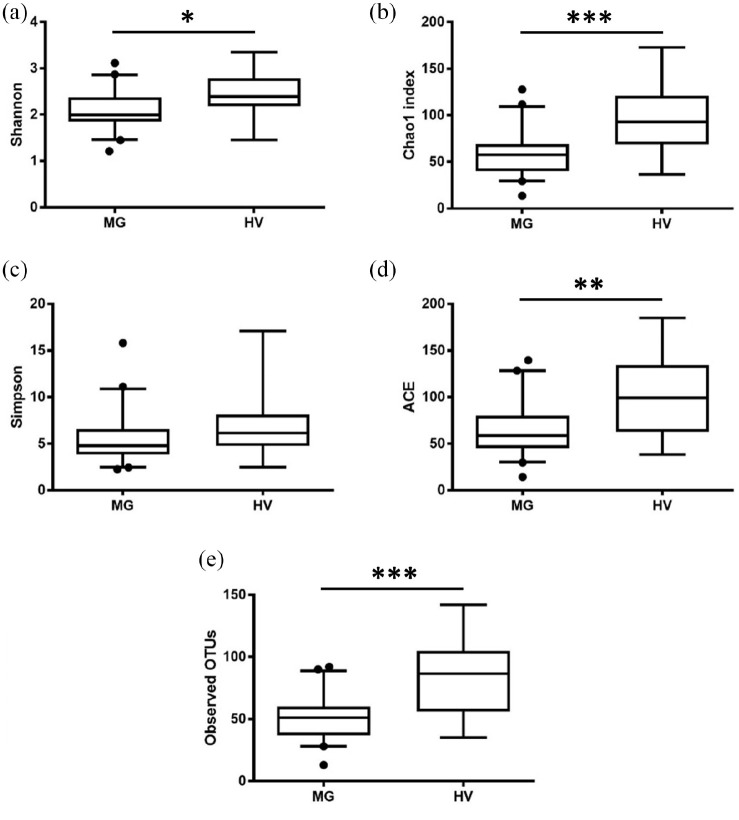
Comparison of alpha-diversity indices of the MG and HV groups using the Mann–Whitney test. Data are presented as mean ± standard error of the mean: (a) *p* = 0.0165; (b) *p* = 0.0007; (d) *p* = 0.0061; (e) *p* = 0.0006. ACE, abundance-based coverage estimator; HV, healthy volunteer; MG, myasthenia gravis; OTU, operational taxonomic unit.

To assess differences in the gut microbiota of patients with MG (*n* = 41), CIDP (*n* = 6) and NIND (*n* = 18) for the primary endpoint, ecological features of the faecal bacterial communities were evaluated using a variety of indices based on the OTU level (Figure 1). The alpha diversity, including observed OTUs, Shannon, Simpson, ACE and Chao1 indices, was not significantly different (*p* > 0.05) between the MG, CIDP and NIND groups. For the secondary endpoint, significant differences were found between patients with MG and healthy volunteers (Figure 2).

**Figure 3.**
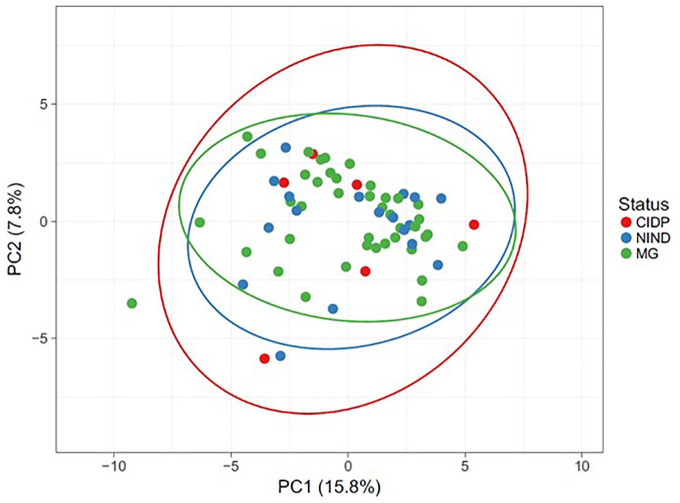
Beta-diversity analysis of bacterial communities using PCA based on log+1 transformed OTU data and PERMANOVA analysis of the weighted UniFrac distance. CIDP, chronic inflammatory demyelinating polyradiculoneuropathy; MG, myasthenia gravis; NIND, non-inflammatory neurological disorder; OTU, operational taxonomic unit; PC1, first principal components; PC2, second principal components; PCA, principal component analysis; PERMANOVA, permutational multivariate analysis of variance.

To estimate the similarity (beta diversity) of microbial community structure between groups, PCA was performed based on OTU profile and log +1 transformed read data. PCA did not cluster samples into distinct groups (Figure 3). The first and second principal components (PC1 and PC2) captured 15.8% and 7.8% of the variation in microbial diversity, respectively. A PERMANOVA analysis of the weighted UniFrac distances for the three groups did not show statistical differences among them.

### Composition of faecal microbiota

**Figure 4.**
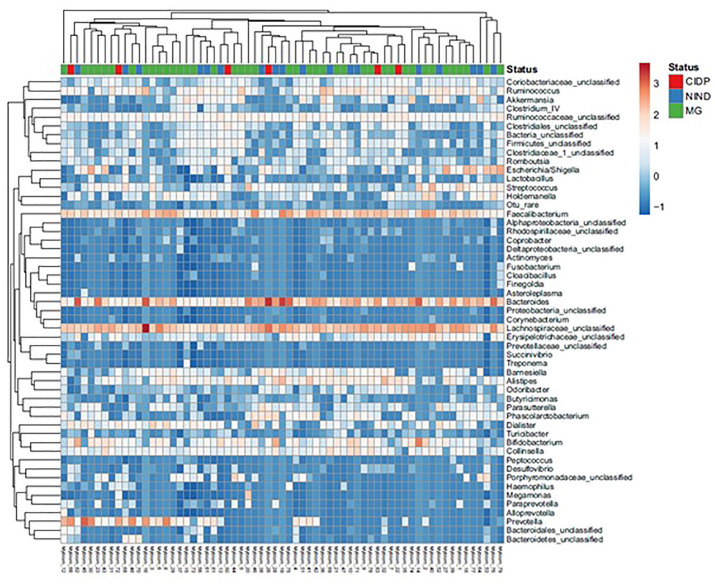
Hierarchical Ward-linkage clustering of faecal samples based on the Spearman distance of the abundance of bacterial genera co-occurrence clusters in each sample. Genus abundance is illustrated by a change in the intensity of colour, with blue to red reflecting low to high abundance. CIDP, chronic inflammatory demyelinating polyradiculoneuropathy; MG, myasthenia gravis; NIND, non-inflammatory neurological disorder.

**Figure 5.**
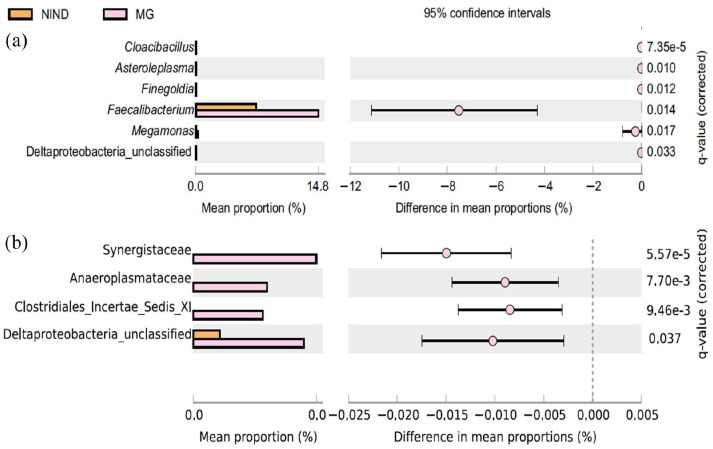
Differentially abundant genera (a) and families (b) in faecal samples from patients with NINDs and MG from a pairwise comparison using Welch’s *t* test with 95% confidence intervals. MG, myasthenia gravis; NIND, non-inflammatory neurological disorder.

**Figure 6.**
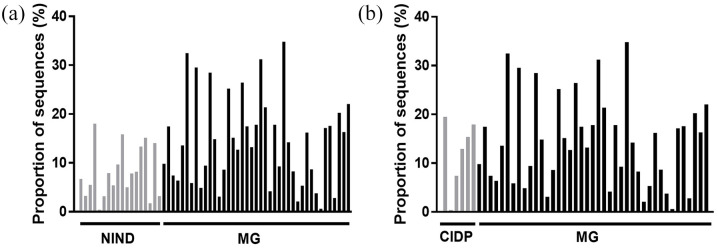
Proportions of *Faecalibacterium* sequences in patients with NINDs *versus* MG (a) and patients with CIDP *versus* MG (b) (CIDP *versus* MG, *p* = 0.5710). CIDP, chronic inflammatory demyelinating polyradiculoneuropathy; MG, myasthenia gravis; NIND, non-inflammatory neurological disorder.

Gut microbiota composition of patients with MG, CIDP and NIND showed no separation based on Spearman distance and Ward hierarchical clustering (Figure 4), although a predominantly positive relationship was observed between the abundance of the three genera *Faecalibacterium*, *Bacteroides* and unclassified *Lachnospira* and samples from all three groups. In a pairwise comparison, the genus *Faecalibacterium* was more abundant in patients with MG than in those with NINDs (*p* = 0.014) (Figures 5(a) and 6(a)). No differences in bacterial genera (data not shown), including *Faecalibacterium* (Figure 6(b)), were detected in faecal samples of MG compared with CIDP. At the family level, unclassified Deltaproteobacteria showed a difference in mean proportions (*p* = 0.037) (Figure 5(b)). Taking corticosteroid treatment in patients with MG into account, a subgroup analysis revealed no difference between *Faecalibacterium* or Deltaproteobacteria (*p* = 1.249 and *p* = 0.883, respectively) (Supplemental Figure 1(a) and (b)).

## Discussion

To the best of our knowledge, this is the first study investigating the intestinal microbiota composition in patients with MG in comparison with three different groups comprising patients with NINDs, CIDP and neurologically healthy individuals in a European cohort. The groups were chosen to allow comparison of gut microbiota among patients with inflammatory disorders of the peripheral nervous system who have different underlying immunological drivers (MG *versus* CIDP) and to compare patients with inflammatory *versus* non-inflammatory disorders (MG *versus* NINDs). Both MG and CIDP are autoimmune diseases of the peripheral nervous system in which a reduction in Treg cells has been described.^26,27^ CIDP is a rare acquired immune-mediated inflammatory disorder. A subgroup of patients with a distinct clinical presentation was found to be positive for antibodies against nodal or paranodal epitopes.^28^ The results provided evidence that Deltaproteobacteria and *Faecalibacterium* are more abundant within the faecal microbiota of patients with MG than in those patients with NINDs. However, no differences were observed in alpha and beta diversity, implying an unaltered bacterial diversity and structure of the bacterial community.

The genus *Faecalibacterium* and its species *F. prausnitzii* belong to the phylum Firmicutes and is one of the most abundant commensal bacteria of the human gut microbiota. *Faecalibacterium* produces a high proportion of the short-chain fatty acid butyrate in the intestine, which has anti-inflammatory effects resulting from Treg cell activation and upregulation of anti-inflammatory cytokine secretion.^29,30^ Although MG is a disorder characterised by reduced Treg cells, we found a higher relative abundance of *Faecalibacterium* in patients with MG compared with NINDs. Previous findings by Moris *et al*.^12^ found similar results in patients with MG compared with healthy volunteers. This finding might be related to MG treatment, especially with corticosteroids. Still, in human oral steroid users, an increase was only identified in *Methanobrevibacter smithii*.^31^ However, corticosteroid treatment (1.0 mg/kg daily for 14 days) had no effect on either the bacterial diversity or composition of gut microbiota in dogs.^32^ To address potential effects on corticosteroid treatment on gut microbiota composition,^33^ we performed a subgroup analysis of patients with MG with and without corticosteroid treatment and found no differences in *Faecalibacterium* and Deltaproteobacteria.

Another finding of our study, which is congruent with Moris *et al*.,^12^ is the increased relative abundance of the class Deltaproteobacteria and the genus *Desulfovibrio*, belonging to this class. Deltaproteobacteria comprise strictly anaerobic genera, which contain many sulphate- (e.g. *Desulfovibrio piger*) and sulphur-reducing bacteria. Granzyme B (GrB) is a proteolytic enzyme present in cytolytic T cells and natural killer cells. Enteric bacteria induce GrB from human colonic natural killer cells and innate lymphoid cells^34^ and its ability to selectively cleave subunits of AChR suggests that GrB-mediated cleavage of AChR exposes cryptic antigens causing an autoimmune response, resulting in a loss of functional AChR.^35,36^ GrB is endocytosed by a mannose 6-phosphate receptor, and binding to this receptor is enhanced by cell surface heparan sulphate.^37^ One can propose a higher availability of sulphate after GrB-mediated apoptosis and ideal conditions for sulphate-reducing bacteria such as Deltaproteobacteria.

The alpha-diversity indices Shannon, Simpson, Chao 1 and ACE were significantly reduced in patients with MG compared with healthy volunteers, implying an altered microbial diversity. These findings correspond to previous studies that also found a difference in the gut microbiota of patients with MG compared with healthy participants. However, beta diversity and the abundance of genera were not affected in this comparison.

Besides two studies from China^13,38^ and one study from Spain,^12^ this is the only other study to assess the intestinal microbiota in patients with MG in a White cohort. This observation is of importance given that MG subtype, prevalence and disease onset differ with ethnicity. For example, the age of onset of MG was higher in Whites than in non-Whites,^39^ whereas in Chinese populations, MG appeared in infancy and in juveniles.^40^ Genetic factors influence Asian and White MG cohorts. Qiu *et al.*^13^ included more patients with MG and healthy controls in their study (53 and 50 *versus* 70 and 74, respectively), while Moris *et al*.^12^ included only 10 patients with MG and 10 controls. Our cohort was dominated by men (female-to-male ratio = 0.71), whereas generally, the female-to-male ratio of patients with MG is approximately 2:1 and varies with age and/or disease classification. Our ratio is comparable to that of Qui *et al*.^13^ (0.71 *versus* 0.71), and the ratio was highest in the study by Moris *et al*.^12^ This must be considered as a factor contributing to microbiota composition when comparing the above-mentioned microbiota studies in patients with MG.

Physical disruption by bead-beating provides sufficient coverage of the original community as well as adequate quality and quantity.^41^ Among the studies mentioned above, only our study used this procedure.

As indicated by recent studies investigating other autoimmune neurological diseases like multiple sclerosis (MS), the gut microbiome may play a crucial role in the pathogenesis of MS.^42,43^ Microbiota alterations affect functions of T-cell populations with an effect on the severity of neurological dysfunctions in mice.^44^ Depending on the type and stages of MS, significant changes in abundance of bacterial species could be observed, for example reduced abundances of *Clostridia* XIVa and IV clusters in the relapsing-remitting form of MS.^45^ In contrast to Takewaki *et al*.,^45^ in our cohort an increase in mean proportion of Clostridiales Incertae Sedis XI was observed in MG *versus* NINDs and, particularly in patients with MG on corticosteroid treatment. This case-control study provided information which can be potentially incorporated into meta-analyses for comparison of (related) auto-immune diseases to identify disease-specific and shared responses.^46^

Overall, the role of antibiotics in exacerbating symptoms of MG should be characterised in larger studies to delineate their effects on neuromuscular transmission from their effects on gut microbiota. An antibiotic treatment of *Faecalibacterium* could be an interesting approach in future randomised controlled interventional trials of MG.

Our study had some limitations. First, corticosteroid intake in more than half of the MG population in our study may influence microbiota and, therefore, the differences observed. Second, the mean age of the MG group was slightly higher than that of the other groups (NIND, CIDP and healthy controls). Comorbidities such as diabetes mellitus may also have an impact on microbiota composition; however, such comorbidities were equally frequent in all groups in our study, thus reducing possible interference. In healthy controls, thyroid disease was interestingly more present than in the MG group.^47^ It is possible that this is due to the smaller size of the healthy volunteer group, the younger age, and the higher percentage of female participants compared with the MG group. NIND group members had quite different neurological disorders. However, the heterogeneity of the NIND group may help to reduce undesired group phenomena and bias potential.

## Conclusion

The MYBIOM study provided evidence that gut microbiota should be considered as a contributor to disease onset and progression in patients with MG. Potential therapeutic approaches modulating the gut microbiota need to be explored in future studies to better understand the involvement of the gut microbiota in the development of this debilitating acquired autoimmune disease.

## Supplemental Material

sj-jpg-1-tan-10.1177_17562864211035657 – Supplemental material for Gut bacterial microbiota in patients with myasthenia gravis: results from the MYBIOM study Click here for additional data file. Supplemental material, sj-jpg-1-tan-10.1177_17562864211035657 for Gut bacterial microbiota in patients with myasthenia gravis: results from the MYBIOM study by Andreas Totzeck, Elakiya Ramakrishnan, Melina Schlag, Benjamin Stolte, Kathrin Kizina, Saskia Bolz, Andreas Thimm, Mark Stettner, Julian R. Marchesi, Jan Buer, Christoph Kleinschnitz, Hedda Luise Verhasselt and Tim Hagenacker in Therapeutic Advances in Neurological Disorders
