# Supplementary figures and images for: Gut bacterial microbiota in patients with myasthenia gravis: results from the MYBIOM study
Source: Ther Adv Neurol Disord. 2021 Aug 11;14:17562864211035657. doi: 10.1177/17562864211035657 (PMC8361534; doi:10.1177/17562864211035657)

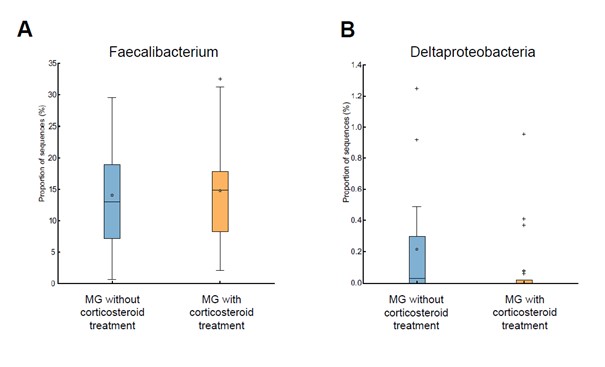

Supplement: sj-jpg-1-tan-10.1177_17562864211035657 – Supplemental material for Gut bacterial microbiota in patients with myasthenia gravis: results from the MYBIOM study [file sj-jpg-1-tan-10.1177_17562864211035657.jpg]
